# Tuning Single-Atom Dopants on Manganese Oxide for Selective Electrocatalytic Cyclooctene Epoxidation

**DOI:** 10.1021/jacs.2c04711

**Published:** 2022-09-13

**Authors:** Minju Chung, Kyoungsuk Jin, Joy S. Zeng, Thu N. Ton, Karthish Manthiram

**Affiliations:** † Department of Chemical Engineering, Massachusetts Institute of Technology, Cambridge, Massachusetts 02139, United States; ‡ Department of Chemistry, Korea University, Seoul 02841, Republic of Korea; § Division of Chemistry and Chemical Engineering, California Institute of Technology, Pasadena, California 91125, United States

## Abstract

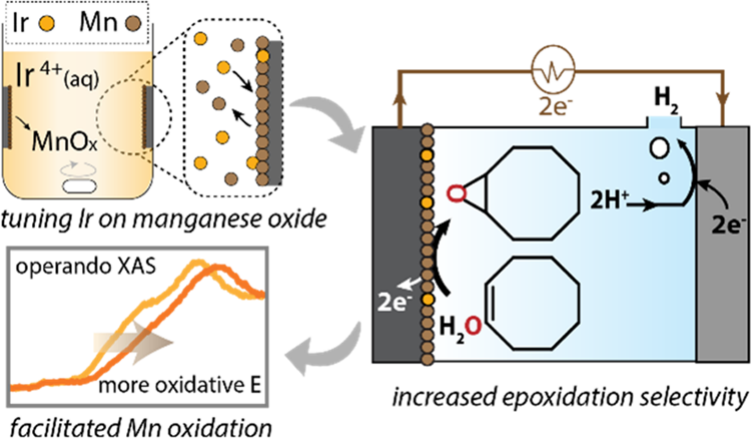

Selective and efficient electrocatalysts are imperative for the successful deployment of electrochemistry toward synthetic applications. In this study, we used galvanic replacement reactions to synthesize iridium-decorated manganese oxide nanoparticles, which showed a cyclooctene epoxidation partial current density of 10.5 ± 2.8 mA/cm^2^ and a Faradaic efficiency of 46 ± 4%. Results from operando X-ray absorption spectroscopy suggest that manganese leaching from the nanoparticles during galvanic replacement introduces lattice vacancies that make the nanoparticles more susceptible to metal oxidation and catalyst reconstruction under an applied anodic potential. This results in an increased presence of electrophilic oxygen atoms on the catalyst surface during reaction conditions, which may contribute to the enhanced electrocatalytic activity toward cyclooctene epoxidation.

## Introduction

The electrification of chemical reactions is an emerging strategy to reduce carbon emissions in the chemical industry. While thermodynamic analyses demonstrate that an electrical potential can efficiently drive various chemical reactions under mild conditions,^1^ achieving high selectivity and activity toward a target reaction remains challenging. For the broader implementation of electricity-driven chemical synthesis, the discovery of high-performance electrocatalysts is critical.

Olefin epoxidation is a crucial chemical functionalization reaction that produces key chemical intermediates for the synthesis of various commercial end products.^2,3^ For example, propylene oxide is produced via the chlorohydrin process, and ethylene oxide is chiefly synthesized using molecular oxygen and silver catalysts. In addition to these two processes, homogeneous catalysts containing terminal metal-oxo species have been reported as epoxidation catalysts.^4−6^ Metal-oxo species generated by peroxide-based oxidants or photoirradiation can provide oxygen atoms to olefin substrates to make epoxide or ketone products. Although these routes have exhibited a high selectivity and yield, there is a need to improve upon these efforts to circumvent elevated temperatures and pressures, undesirable stoichiometric byproducts, explosive peroxide-based oxidants,^7^ and high catalyst separation costs. In this regard, a heterogeneous electrochemical process that can directly epoxidize olefins under ambient conditions presents an attractive alternative to the existing epoxidation routes.

Several research groups have recently attempted olefin epoxidation via electrochemical methods. In situ electrochemical generation of chemical oxidants such as hydrogen peroxide,^8−10^ active halogens,^11,12^ or peroxodicarbonate^13^ were used to convert olefin substrates to their corresponding epoxides. Our group previously reported new electrochemical routes for olefin epoxidation, where sub-10-nm-sized manganese oxide nanoparticles catalyzed the direct epoxidation of cyclooctene using water as an oxygen atom source, with a faradaic efficiency of ∼30%.^14^ Based on electrochemical kinetic studies, the generation of Mn(IV)=O species was suggested to be the resting state of the catalytic cycle, facilitating the transfer of the oxygen atom to the cyclooctene substrate. While this method provides an environmentally friendly and safe route to make epoxides, its low faradaic efficiency and yield must be improved for it to become industrially relevant.

One way to improve the efficiency of heterogeneous catalysts is through the introduction of atomically dispersed metal atoms on the appropriate supporting materials. These catalysts with atomically dispersed metal atoms have exhibited enhanced specific activity and high selectivity due to their unsaturated coordination environment, which facilitates their ability to act as active sites and achieve unexpected selectivity.^15−17^ For catalysts where the isolated atoms act as the sole active site, arranging as many isolated atoms as possible on the substrate is desirable to maximize atom economy.^18^ On the other hand, single metal centers can also be introduced to a substrate that already acts as a catalyst for the target reaction. In this case, it is important to consider the geometric and electronic tuning of the original active sites upon the introduction of guest atoms to the host catalyst, in addition to their role as additional active sites. In this vein, we used a galvanic replacement method to attain single to cluster iridium atoms decorated on manganese oxide catalysts while generating manganese vacancies. This modification increases the selectivity of both manganese and iridium oxide catalysts toward olefin epoxidation. Herein, we report a new catalyst, single iridium-decorated manganese oxide nanoparticles (Ir_single_-MnO*_x_* NPs), which exhibited a nearly 50% faradaic efficiency for cyclooctene epoxidation. Furthermore, a series of electrochemical kinetic studies and operando X-ray absorption spectroscopy (XAS) analyses provided insights into the structure–activity relationship of cyclooctene epoxidation by Ir_single_-MnO*_x_* NPs.

## Results and Discussion

### Synthesis of Ir-MnO*_x_* NPs Using Galvanic Replacement Reaction

**Figure 1:**
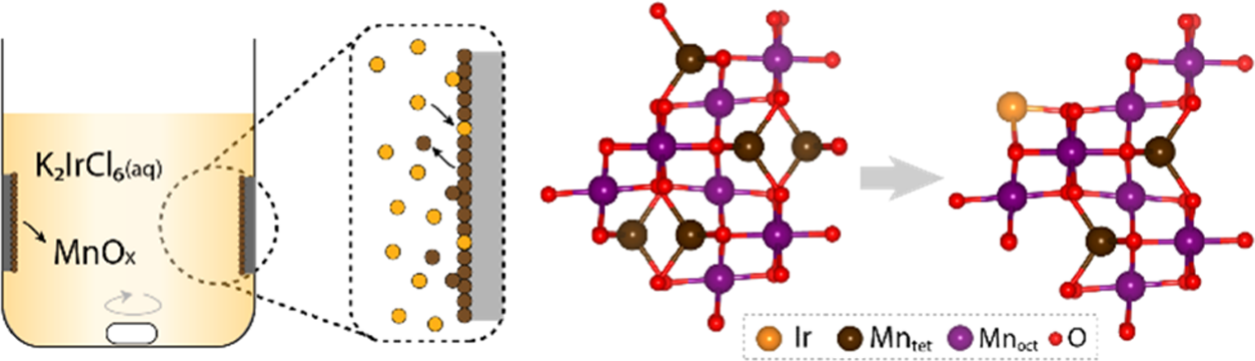
Scheme of the galvanic replacement between the iridium precursor and manganese oxide catalyst.

The MnO*_x_* NPs of the Mn_3_O_4_ (hausmannite) phase were prepared via hot injection,^14^ and iridium atoms were decorated on the surface of the nanoparticles by a galvanic replacement reaction (see the Experimental Section and Supporting Information for details). The galvanic reaction step involved a spontaneous redox reaction between K_2_IrCl_6_ and MnO*_x_* nanoparticles deposited on a carbon paper substrate; simultaneous dissolution of manganese atoms and deposition of iridium heteroatoms on the surface are driven by the difference in the redox potential between the two metals involved.^19,20^ Although the exact driving force varies depending on the local concentration of participating species and the details on the coordination environment on the surface, the standard electrode potential of the metals was used as a baseline for predicting the probability of a given pairing of metals toward galvanic replacement. The standard reduction potential of the iridium precursor (eq 1) is 0.835 V, while the reduction potential of the phase transition between β-MnO_2_ and Mn_3_O_4_ was calculated to be 0.555 V (eq 2; see also Table S1).^21^ The higher reduction potential of the iridium precursor allows for the spontaneous reduction of IrCl_6_^2–^ coupled with the oxidation of Mn_3_O_4_ (eq 3), which drives the deposition of iridium on the manganese oxide surface and the concomitant leaching of manganese (Figure 1).
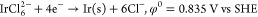
1
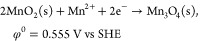
2
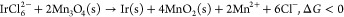
3

### Catalyst Characterizations

**Figure 2:**
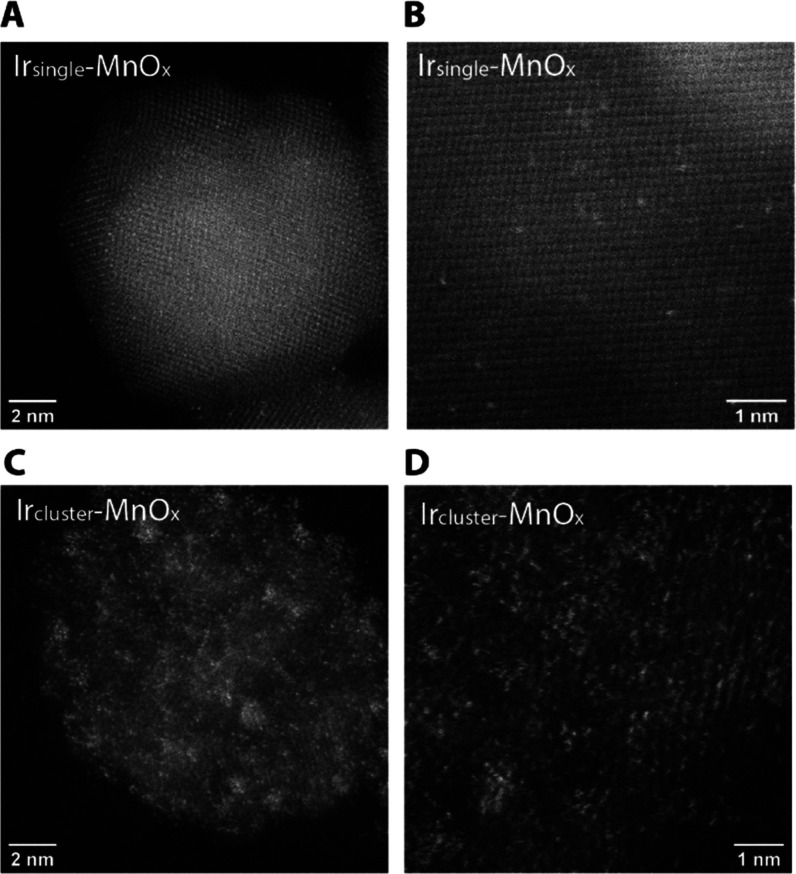
HAADF-STEM images of (A, B) Ir_single_-MnO*_x_* and (C, D) Ir_cluster_-MnO*_x_*.

The Ir-MnO*_x_* NPs were characterized using high-angle annular dark-field (HAADF) imaging with aberration-corrected scanning transmission electron microscopy (STEM). Incoherent Z-contrast imaging and high spatial resolution allowed the determination of the iridium atom distribution on the manganese oxide nanoparticle supports.^22^ Under mild synthetic conditions, single iridium atoms were randomly dispersed in the MnO*_x_* lattice, appearing as brighter spots in the STEM images (Ir_single_-MnO*_x_*; Figure 2A,B). The iridium loading was controlled by adjusting the concentration of the precursor and the temperature of the galvanic replacement reaction. Upon increasing the galvanic replacement reaction time, the concentration of the iridium precursor, and the reaction temperature, the loading of iridium atoms on the Ir-MnO*_x_* surface also increased, leading to the formation of clusters (Figures S4 and 2C,D). The size and the number of clusters increased in the following order: Ir_few_-MnO*_x_* < Ir_few/cluster_-MnO*_x_* < Ir_cluster_-MnO*_x_* (see Section A.2. for details).

**Figure 3:**
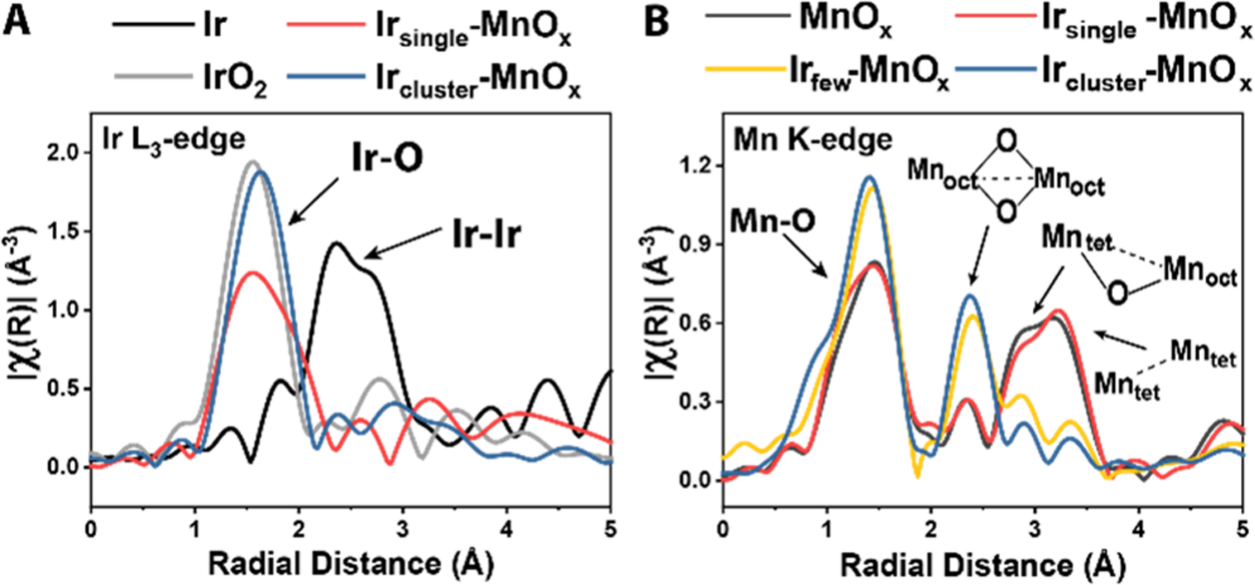
Fourier transform EXAFS spectra of Ir-MnO*_x_* samples at (A) the Ir L_3_-edge and (B) the Mn K-edge. Note that the radial distance (scattering length) is ∼0.5 Å shorter than the bond length between the scatterers.

We then used XAS to probe the oxidation state and local coordination environment of the metals in the synthesized Ir-MnO*_x_*, which encompasses samples ranging from single atoms (Ir_single_-MnO*_x_*) to clusters (Ir_cluster_-MnO*_x_*) (Figure 3). The extended X-ray absorption fine structure (EXAFS) at the Ir L_3_-edge suggests that the short-range scattering features of Ir-MnO*_x_* resemble those of IrO_2_, indicating that the iridium atoms are surrounded by oxygen for samples containing single atoms and clusters of iridium alike. The prominent peak at ∼1.6 Å corresponds to the Ir–O scattering path, suggesting that iridium is coordinated by oxygen in Ir-MnO*_x_*, which by itself might imply a local coordination environment similar to that of IrO_2_. However, the second and higher shells of Ir-MnO*_x_* do not match IrO_2_, which could indicate scattering paths from Ir–Mn instead of Ir–Ir. Furthermore, the lower intensity of the Ir–O peak in Ir_single_-MnO*_x_* compared to IrO_2_ or Ir_cluster_-MnO*_x_* implies that iridium is undercoordinated in Ir_single_-MnO*_x_*. The estimated coordination number from the EXAFS fitting for iridium in Ir_single_-MnO*_x_* was 5 ± 1, while Ir in IrO_2_ has a coordination number of 6.

Compared to Ir_single_-MnO*_x_*, Ir_cluster_-MnO*_x_* shares more similarities with IrO_2_, which can be ascribed to another pair of galvanic reactions that oxidize iridium on the catalyst surface. For Ir_single_-MnO*_x_*, the ICP-OES analysis (Table S2) of the postgalvanic replacement solution showed that the amount of consumed iridium precursor was comparable to the amount of manganese leached out. However, for Ir_cluster_-MnO_*x*,_ excess iridium was consumed from the solution relative to the amount of manganese that dissolved into the solution. The result suggests that the deposition of iridium beyond a certain point does not require manganese dissolution. Instead, the iridium deposition on the catalyst can be galvanically coupled with the oxidation of the iridium clusters, which are essentially combined as the hydrolysis of the iridium precursor on the MnO*_x_* surface (eq 5). It is worth noting that this favorable reaction does not imply that we should expect a well-defined crystalline IrO_2_ phase on the surface. The reaction implies that the deposited iridium atoms have a tendency to be oxidized, forming bonds with neighboring oxygen atoms rather than remaining in a more reduced form.
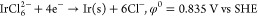
1
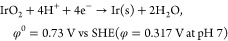
4
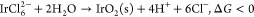
5Manganese leaching during galvanic replacement generated lattice vacancies, which increased the average Mn oxidation state in the nanoparticles (Figure S5). The average manganese oxidation states estimated from the X-ray absorption near-edge structure (XANES) followed the order: Mn_3_O_4_ < MnO*_x_* < Ir_single_-MnO*_x_* < Ir_few_-MnO*_x_* ≈ Ir_cluster_-MnO*_x_* < MnO_2_. A higher manganese oxidation state is correlated with a shortened Mn–O bond in Ir_few_-MnO*_x_* and Ir_cluster_-MnO*_x_* compared to that in MnO*_x_* and Ir_single_-MnO*_x_*, as shown by EXAFS at the Mn K-edge (Figure 3B). The EXAFS further revealed that pristine MnO*_x_* and Ir_single_-MnO*_x_* both have Mn_3_O_4_-like structures, characterized by a Mn–Mn scattering path (*R*_Mn(oct)-Mn(tet)_ = 3.495 Å) from corner-sharing octahedral Mn and tetrahedral Mn. In contrast, Ir_few_-MnO*_x_* and Ir_cluster_-MnO*_x_* exhibit MnO_2_-like structures, showing a shorter Mn–Mn scattering path (*R*_Mn(oct)-Mn(oct)_ = 2.93 Å) resulting from edge-sharing octahedral Mn atoms. This implied that the manganese oxidation state increased as the galvanic replacement reaction progressed, and the highly oxidized Ir-MnO*_x_* samples underwent reconstruction from a Mn_3_O_4_-like structure to a MnO_2_-like structure.

The EXAFS fitting of the Mn K-edge was performed to probe the structural differences between MnO*_x_* and Ir_single_-MnO*_x_*. The first shells of the samples were fitted to the scattering path of Mn_3_O_4_. A linear combination fitting of the tetrahedral and octahedral Mn–O scattering paths on the first shells of MnO*_x_* and Ir_single_-MnO*_x_* was performed to estimate the proportion of Mn at the tetrahedral site. The ratios of the Mn tetrahedral site in Ir_single_-MnO*_x_* (0.2 ± 0.1) and MnO*_x_* (0.3 ± 0.1) are within the errors of each other (Table S3). For the Ir_few_-MnO*_x_* and Ir_cluster_-MnO*_x_* samples that were treated with a higher extent of galvanic replacement, Mn_tet_–Mn_oct_ and Mn_tet_–Mn_tet_ peaks in FT-EXAFS were diminished (Figure 3B). These results suggest that Mn(II) in tetrahedral sites may be liberated from the catalyst in exchange for iridium during galvanic deposition.

### Electrochemical Kinetic Study

**Figure 4:**
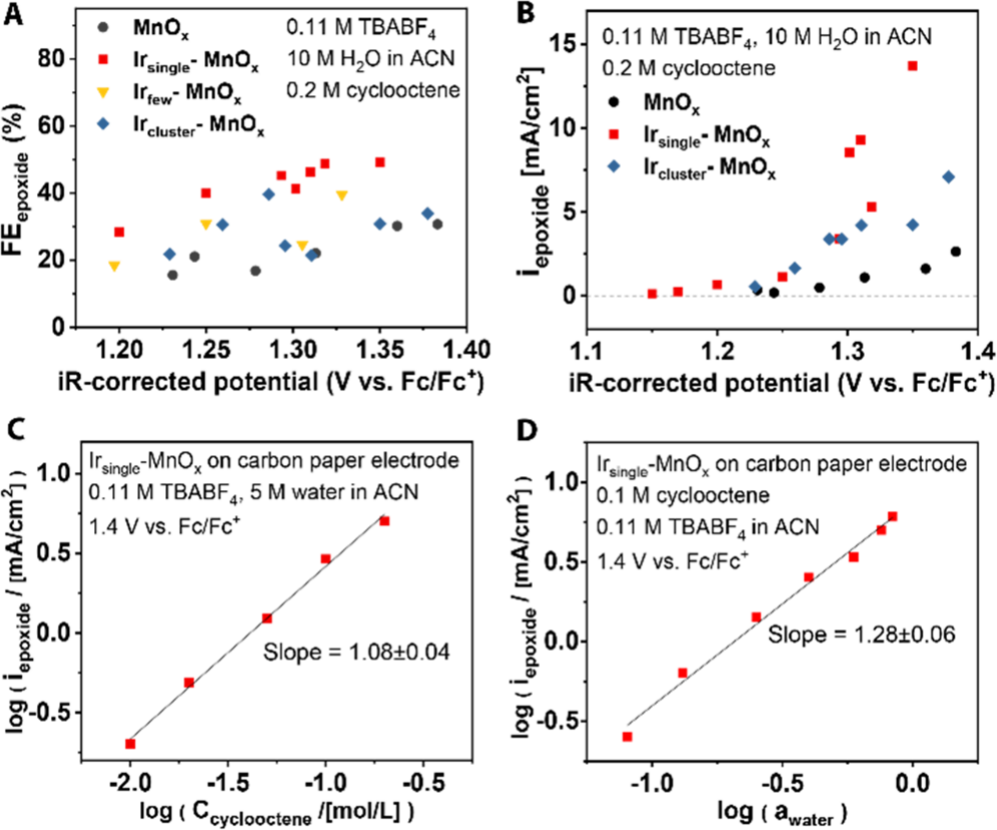
Ir-MnO*_x_* catalysts for cyclooctene epoxidation. (A) Faradaic efficiency for cyclooctene epoxidation vs potentials. (B) Comparison of average epoxidation current between Ir_single_-MnO*_x_*, Ir_cluster_-MnO*_x_*, and MnO*_x_*. (C) Cyclooctene concentration (at 5 M H_2_O) and (D) water concentration (at 100 mM cyclooctene) dependences of average epoxide partial current at 1.4 V vs Fc/Fc^+^. Acetonitrile (ACN) was used as the solvent.

Ir_single_-MnO*_x_* showed higher selectivity and activity for cyclooctene epoxidation than pristine MnO*_x_* (Figure 4A,B). Electrochemical kinetic studies were conducted by chronoamperometry at varying potentials and substrate concentrations. In a typical experiment, 10 C of charge was passed, which was equivalent to a maximum conversion of ∼6.5% of the substrate. The partial current density toward epoxidation was higher with Ir_single_-MnO*_x_* than with pristine MnO*_x_*, showing an especially large gap at potentials above 1.3 V vs Fc/Fc^+^. Compared with MnO*_x_*, the epoxidation rate increased more rapidly with Ir_single_-MnO*_x_* in response to the applied potential. Rate law analysis conducted using Ir_single_-MnO*_x_* NPs showed a first-order dependence on the cyclooctene concentration and water activity (Figure 4C,D). These results are consistent with the mechanism proposed for the epoxidation of olefins by Mn_3_O_4_-based NP catalysts (see Figure S6 and relevant discussion in Supporting Information Section E).^14^ In our previous work, we proposed Mn(IV)=O as the reactive intermediate that transfers the oxygen atom to the olefin substrate, leaving Mn(II)-vacant sites. Considering the nucleophilic nature of the carbon–carbon double bond in cyclooctene, increasing the electrophilicity of the oxygen atom on the catalyst may facilitate epoxidation.^23,24^ As discussed earlier, XAS analysis showed that the average oxidation state of manganese in MnO*_x_* increased after iridium decoration and the introduction of manganese vacancies via galvanic replacement. The increase in the formal oxidation state of manganese might increase the electrophilic character on the oxygen ligands by the induced hole-doping effect.^25^ This might explain why Ir_single_-MnO*_x_* showed more selective epoxidation capability than pristine MnO*_x_*.

However, Ir-MnO*_x_* with higher iridium loadings was not as selective as Ir_single_-MnO*_x_* for cyclooctene epoxidation. Notably, Ir_single_-MnO*_x_* exhibited a distinct structure from Ir_few_- or Ir_cluster_-MnO*_x_*. Ir_single_-MnO*_x_* can be described as a Mn_3_O_4_-like structure decorated with unclustered iridium atoms on its surface. In contrast, Ir_few_- and Ir_cluster_-MnO*_x_* contained aggregated iridium atoms on their surfaces. Clusters of oxidized iridium on the surface can provide active sites that are more selective toward oxygen evolution than epoxidation since iridium oxides are well-established water oxidation catalysts.^26^ Iridium oxide nanoparticles exhibited lower epoxidation selectivity than MnO*_x_* or Ir-MnO*_x_* catalysts with an FE_epoxide_ = 25 ± 3% (*n* = 2) at 1.45 V vs Fc/Fc^+^ (85% *iR*-compensated) using 0.2 M cyclooctene and 10 M H_2_O.

We have investigated other heteroatom-decorated metal oxide nanocatalysts to find out if there are better combinations for epoxidation. The galvanic replacement was also performed using other heteroatoms and supporting metal oxide catalysts. Pt_single_-MnO*_x_* was synthesized with K_2_PtCl_6_ instead of K_2_IrCl_6_ (Figure S7A), but the FE_epoxide_ did not increase significantly (from 25 to 30% for MnO*_x_* to 33% for Pt_single_-MnO*_x_*). When FeO*_x_* was used instead of MnO*_x_*, atomic iridium was successfully dispersed on FeO*_x_* (Figure S7B), but the modification did not result in any improvement in the epoxidation selectivity (FE_epoxide_ = 12%) or activity (Table S6). A specific combination of iridium and MnO*_x_* was required to achieve enhanced epoxidation selectivity upon decorating the base metal oxide with single atoms.

### Operando XAS at Mn K-Edge and Ir L_3_-Edge

**Figure 5:**
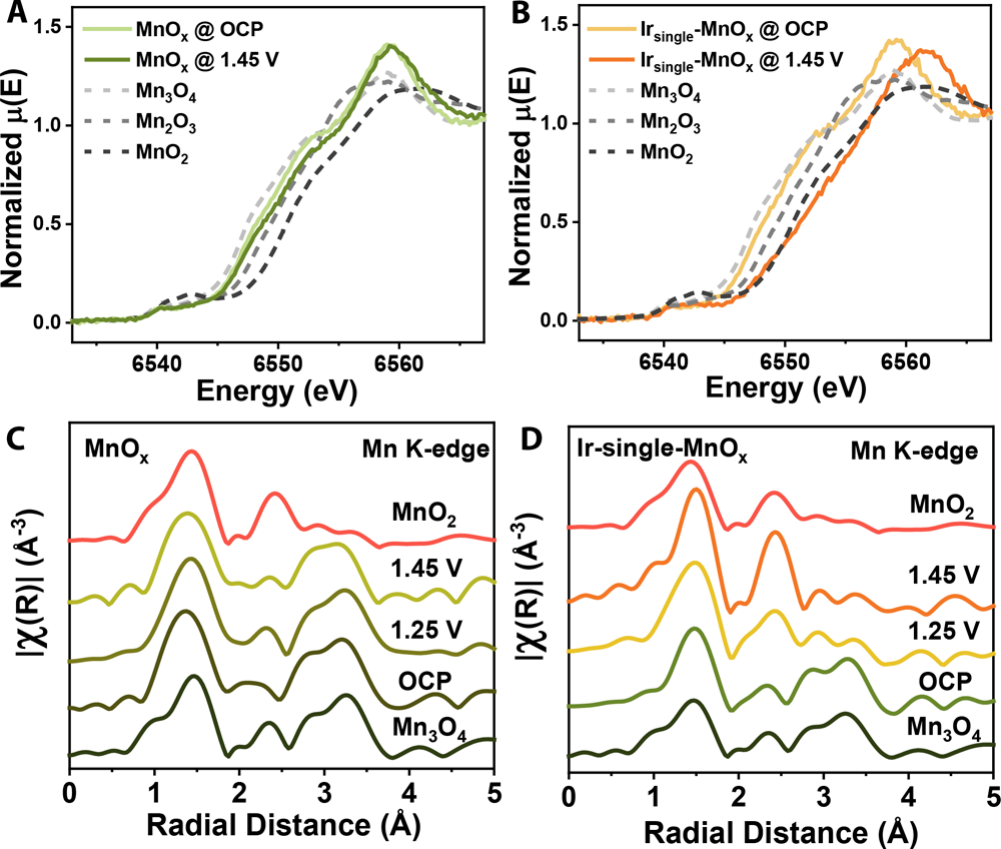
Operando XAS at the Mn K-edge. Shifts in XANES spectra for (A) MnO*_x_* and (B) Ir_single_-MnO*_x_* catalysts. Fourier transform EXAFS spectra for (C) MnO*_x_* and (D) Ir_single_-MnO*_x_* catalysts. The potentials are 85% *iR*-compensated.

To directly probe the relationship between the catalyst properties and performance, operando XAS at the Mn K-edge and Ir L_3_-edge was conducted. The manganese oxidation state under anodic bias increased more dramatically after single iridium atoms were deposited on the surface of MnO*_x_* (Figure 5A,B). This result implies facile oxidation of manganese when MnO*_x_* is decorated with iridium to form Ir_single_-MnO*_x_*, generating the electrophilic oxygen species that participate in epoxidation. Moreover, we tracked the manganese coordination environment in MnO*_x_* and Ir_single_-MnO*_x_* under epoxidation conditions with increasing anodic potential (Figure 5C,D). Although MnO*_x_* remained in its Mn_3_O_4_-like structure throughout the entire experiment, Ir-MnO*_x_* transformed from a Mn_3_O_4_-like structure into a MnO_2_-like structure as the applied anodic potential increased. In Mn_3_O_4_, tetrahedral Mn(III) oxidized from Mn(II) is kinetically trapped to remain as Mn(III), and the oxidation of octahedral Mn(III) is sluggish due to stabilization by Jahn–Teller distortion.^27^ This explains why MnO*_x_* retains its structure and initial oxidation state under increasing anodic potential. Meanwhile, mild tuning of MnO*_x_* with iridium single atoms facilitates manganese oxidation and the associated phase change under anodic potentials.

Structural information also provides insight into the higher performance of Ir_single_-MnO*_x_* compared to Ir_few_-MnO*_x_* and Ir_cluster_-MnO*_x_*. We characterized the catalysts with X-ray photoelectron spectroscopy (XPS) to collect surface-specific information. While a higher extent of galvanic replacement could increase the initial oxidation state of manganese and the electrophilicity of the lattice oxygen atoms, there is a good chance that the metal-depleted oxygen atoms can take up protons, forming hydroxyl groups on the surface. In the case of iridium-decorated MnO*_x_* NPs, we observed a peak at ∼531.5 eV in the O 1s XPS spectrum, corresponding to characteristic surface hydroxyl groups (−OH) (Figure S8). Interestingly, predominant hydroxyl peaks were observed at the spectra of Ir_cluster_-MnO*_x_* compared to those of Ir_single_-MnO*_x_* or MnO*_x_* nanoparticles. Similarly, Pt-MnO*_x_* also exhibited a similar fashion in the O 1s XPS spectrum. Pt_single_-MnO*_x_* showed a higher abundance of hydroxyl species (Figure S9A), and the manganese oxidation state of Pt_single_-MnO*_x_* was higher than that of Ir_single_-MnO*_x_* (Figure S9B). High coverage of surface hydroxyl species has been suggested as a descriptor for an enhanced oxygen evolution reaction (OER) activity.^28^ Considering that the OER is a major competing reaction of cyclooctene epoxidation (Figure S11) and electrophilic oxygen species were believed to be responsible for a higher OER activity,^25^ we believe that the Ir_cluster_-MnO*_x_* and Pt-MnO*_x_* catalysts showed low selectivity toward epoxidation due to the surface hydroxyl species. Therefore, we would like to emphasize that achieving an appropriate degree of manganese oxidation and oxygen electrophilicity, in addition to the lack of iridium clusters on the surface, is important to suppress the OER while achieving an enhanced epoxidation activity.

**Figure 6:**
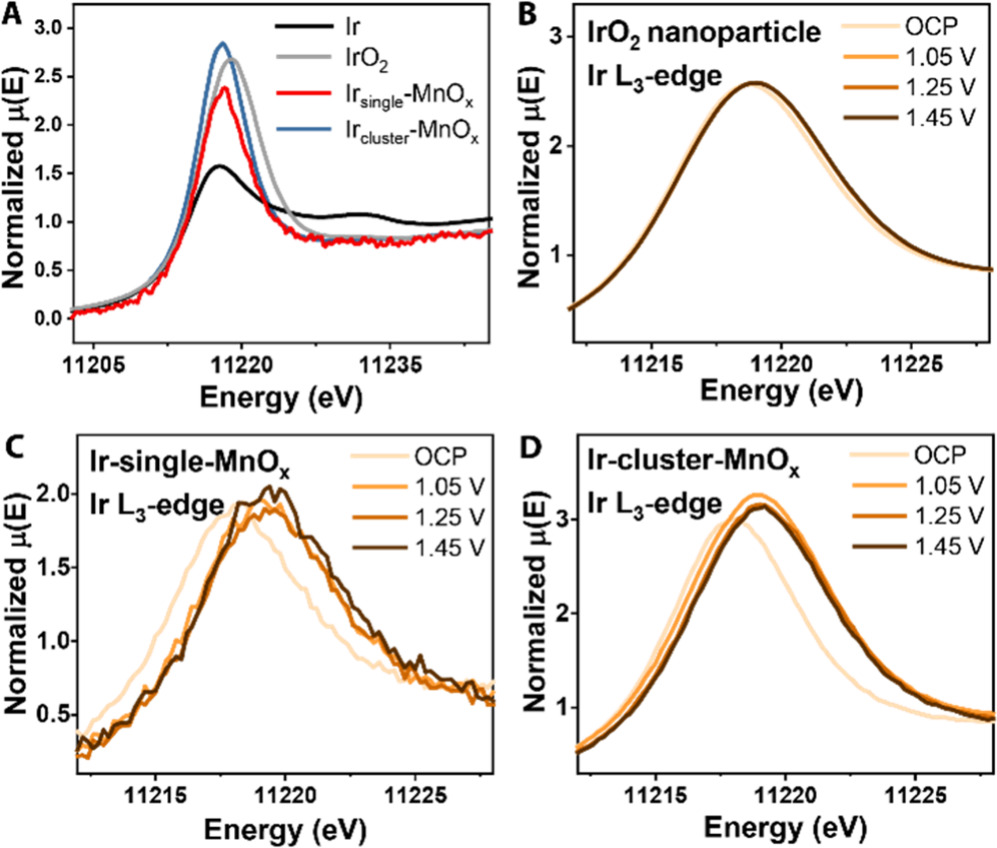
XANES at the Ir L_3_-edge. (A) Comparison of the Ir L_3_-edge white line for iridium reference materials and Ir-MnO*_x_* catalysts. Edge shifts were examined for (B) IrO_2_ nanoparticles, (C) Ir_single_-MnO*_x_*, and (D) Ir_cluster_-MnO*_x_* under applied anodic potential. The potentials are 85% *iR*-compensated.

The oxidation states of iridium in the Ir-MnO*_x_* catalysts can be inferred from the Ir L_3_-edge XANES spectrum, which is characterized by broad white lines corresponding to a transition from occupied 2p to empty 5d states. The higher white line indicates less-occupied d-orbital states and, thus, a lower electron density.^29^ Moreover, the shift of the white line position is proportional to the oxidation state of iridium species in iridium oxides. The white line positions of Ir-MnO*_x_* samples are at lower energy compared to that of IrO_2_, indicating that its iridium oxidation state before applying the potential is lower than +4 (Figure 6A). The lower oxidation state of iridium in Ir_single_-MnO*_x_* is consistent with its longer Ir–O bond lengths compared to that in IrO_2_. The estimated Ir–O bond length from EXAFS fitting was longer for lower Ir loading samples: 1.983 ± 0.006 Å (IrO_2_) < 2.04 ± 0.01 Å (Ir_cluster_-MnO*_x_*) < 2.08 ± 0.02 Å (Ir_single_-MnO*_x_*).

Upon applying an anodic potential, the edge shift and white line increase were not apparent in the IrO_2_ nanoparticle (∼12 nm) catalysts (Figure 6B), presumably due to its small surface-to-bulk ratio. In contrast, the white line positions in Ir-MnO*_x_* samples clearly shifted to higher energy (Figure 6C,D), which indicates that the iridium atoms as well as manganese atoms contributed to making the adjacent oxygen atom more electrophilic by creating electron-poor metal sites.

## Conclusions

We probed the electronic states and local geometric structures of the Ir_single_-MnO*_x_* catalyst under electrochemical cyclooctene epoxidation conditions using operando XAS. The mild galvanic replacement tuning of MnO*_x_* with iridium single atoms enabled dynamic catalyst reconstruction and facile metal oxidation under an anodic potential. Highly electrophilic oxygen atoms induced by adjacent electron-poor metals were possibly responsible for the enhanced electrocatalytic cyclooctene epoxidation performance on Ir_single_-MnO*_x_* compared to undecorated MnO*_x_*. The lower selectivity toward epoxidation with pre-reconstructed Ir_few_-MnO*_x_* and Ir_cluster_-MnO*_x_* catalysts can be attributed to the prevalent surface hydroxyl species and oxidized iridium clusters on the catalyst. Our findings highlight that galvanic replacement reactions can be used for the mild tuning of metal oxide catalysts by introducing heteroatoms as well as by modifying the structural and electronic properties of the catalyst.

## Experimental Section

### Electrode Preparation

Sub-10-nm-sized manganese oxide nanoparticles (MnO*_x_* NPs) were synthesized by hot-injection and deposited on hydrophilic carbon paper (CP) electrodes. A series of Ir-MnO*_x_* NPs were prepared from the deposited MnO*_x_* NPs via the galvanic replacement method. Four MnO*_x_*/CP electrodes were added to the beaker using a Kapton tape. The beaker was filled with an aqueous solution of K_2_IrCl_6_ and placed in a water bath. The temperature, reaction time, and precursor concentration were adjusted to tune the iridium loading on the MnO*_x_* NPs (see the detailed synthesis procedure in the Supporting Information). The prepared electrodes were used as anodes for electrochemical studies.

### Electrochemical Study

A sandwich-type one-compartment cell was used for the electrochemical studies. Platinum foil and Ag/AgCl electrodes acted as the cathode and reference electrodes, respectively. Acetonitrile (ACN) with 0.11 M tetrabutylammonium tetrafluoroborate (TBABF_4_) was used as the electrolyte, with varying concentrations of cis-cyclooctene and water. Potentials were either 100% *iR*-compensated (*i* = current, *R* = resistance) manually or 85% *iR*-compensated automatically based on the initial electrochemical impedance spectroscopy results. After electrolysis, additional water, excess hexane, and an internal standard, 1,3,5-trimethoxybenzene, were added to the electrolyte. Oxidation products dissolved in the electrolyte were extracted into the hexane layer and quantified using nuclear magnetic resonance (NMR) spectroscopy. The gas-phase products, including hydrogen and oxygen, were quantified using on-line gas chromatography (SRI Instruments), with N_2_ gas flowing through the cell as a carrier gas.

### X-ray Absorption Spectroscopy

Operando XAS spectra were collected in X-ray fluorescence mode at beamlines 8-ID (Ir L_3_-edge) and 7-BM (Mn K-edge) of the National Synchrotron Light Source II and 10.3.2 (Mn K-edge) of the Advanced Light Source. The same set of electrochemical cells used for the kinetic studies was used, except for a backplate with a hole for the X-ray entrance. A Kapton polyimide film was placed between the window and electrode to prevent electrolyte leakage (Figure S12). All XAS data were processed using the Athena software for background removal and normalization. The EXAFS data were further modeled and analyzed using the Artemis software. The amplitude reduction factor (*S*_0_^2^) for each metal edge was determined by fitting the reference material with known coordination numbers. The *S*_0_^2^ values were used in other simulations to estimate the coordination numbers of the samples.

## Supporting Information Available

Experimental methods including materials, catalyst, and electrode preparation; TEM analysis; electrochemical methods; product analysis; additional data; and discussions on the mechanism (PDF)
