# Supplementary material for: Tuning Single-Atom Dopants on Manganese Oxide for Selective Electrocatalytic Cyclooctene Epoxidation
Source: J Am Chem Soc. 2022 Sep 13;144(38):17416–22. doi: 10.1021/jacs.2c04711 (PMC9523708; doi:10.1021/jacs.2c04711)
Supplement: Supplementary file 1 — ja2c04711_si_001.pdf [file ja2c04711_si_001.pdf]

Supporting Information

Tuning single atom dopants on manganese oxide for selective electrocatalytic cyclooctene epoxidation

Minju Chung,[1,#] Kyoungsuk Jin,[1,2,#] Joy S. Zeng,[1] Thu N. Ton,[3] Karthish Manthiram[3,*]

[1]Department of Chemical Engineering, Massachusetts Institute of Technology, Cambridge, MA 02139, United States

[2]Department of Chemistry, Korea University, Seoul 02841, Republic of Korea

[3]Division of Chemistry and Chemical Engineering, California Institute of Technology, Pasadena, CA 91125, United States

[#] These authors contributed equally to this work: M.C. and K.J.

[*]Correspondence: karthish@caltech.edu

Contents

A. Materials and Methods 2

A.1. Materials 2

A.2. Catalysis preparation 2

A.3. TEM analysis 4

A.4. X-ray photoelectron spectroscopy 4

B. Electrochemical study 5

B.1. Electrochemical methods 5

B.2. Long-term electrolysis 6

C. Product analysis 6

D. Supplementary Tables and Figures 7

E. Discussion on the cyclooctene epoxidation mechanism 14

References 16

A. Materials and Methods

A.1. Materials

A.1.1. Catalyst preparation

Manganese(III) acetate dihydrate (97%, Sigma Aldrich), octadecene (90%, Sigma Aldrich), 1-decanol (90% Sigma Aldrich), nitrosyl tetrafluoroborate ($NOBF_4$, 95%, Sigma Aldrich), myristic acid (99%, Sigma Aldrich), iron(II) acetate (95%, Thermo Scientific), potassium hexachloroiridate(IV) (K_2IrCl_6, 99%, Sigma Aldrich), potassium hexachloroplatinate(IV) (K_2PtCl_6, 98%, Sigma Aldrich), acetone (99.5%, VWR), toluene (99.5%, VWR), hexane (99.9%, Fisher Scientific), ethanol (Anhydrous, KOPTEC USP), and N,N-Dimethylformamide (DMF, 99.8%, Sigma Aldrich) were purchased and used without additional treatment.

A.1.2. Electrochemical study

cis-cyclooctene (cyclooctene, 95%, Alfa Aesar), cis-cyclooctene oxide (99%, Sigma Aldrich), cyclooctanone (98%, Sigma Aldrich), tetrabutylammonium tetrafluoroborate ($TBABF_4$, 99%, Acros Organics), Acetonitrile (ACN, anhydrous, 99.8+%, Alfa Aesar), 1,3,5-Trimethoxybenzene (TMB, 99 %, Sigma Aldrich), Deuterated chloroform (99.8 atom % D, Sigma Aldrich), Ferrocene(Fc, 99%, Alfa Aesar) were purchased and used without additional treatment. Indium-doped tin oxide (ITO) electrodes were purchased from Delta Technologies. Disk-shaped carbon paper electrodes with a diameter of 14 mm were punched from carbon paper (Toray, TGP-H-060, Fuel Cell Earth LLC).

A.1.3. X-ray absorption spectroscopy reference materials

Manganese(IV) oxide (MnO_2, ≥99%, Sigma Aldrich), Manganese(III) oxide (Mn_2O_3, 99.9%, Sigma Aldrich), Manganese(II,III) oxide (Mn_3O_4, 97%, Sigma Aldrich), Manganese(II) oxide (MnO, 99%, Sigma Aldrich), Iridium (Ir, -22mesh, 99.99%, Alfa Aesar), Iridium(IV) oxide (IrO_2, 99%, Alfa Aesar), were purchased and mixed with poly(ethylene glycol) (average mol wt 10000, Sigma Aldrich) and pressed into pellets for transmission-mode measurements.

A.2. Catalyst preparation

Heteroatom decoration on transition metal oxide nanoparticles (M_1-M_2O_x ; M_1 = Ir, Pt, M_2 = Mn, Fe) were prepared via galvanic replacement method. Diluted $K_2M(IV)Cl_6$ (M: Ir, Pt) solution was used as the heteroatom precursors, and transition metal oxide nanoparticles (MnO_x, FeO_x) were used as supporting materials. In the case of Ir-MnO_x nanocatalyst, various configurations were achieved via varying synthesis temperature and dwelling time, which is described in detail below.

A.2.1. Ir_{single}-MnO_x synthesis

BF_4-treated MnO_x nanoparticles were prepared on a carbon paper electrode (MnO_x/CP) or a ITO glass electrode using a previously described method.[1] Briefly, sub-10-nm-sized MnO_x nanoparticles were synthesized by using hot injection method and ligand exchange was performed with $NOBF_4$ to remove the organic ligand on the manganese oxide nanoparticle. Hydrophilic carbon paper electrodes were prepared by heating 14-mm diameter hydrophobic carbon papers in a muffle furnace at 600 °C for 1 hour. Next, 15 μL of ligand-exchanged MnO_x nanoparticles in ethanol solution were dropped on the hydrophilic carbon paper electrode in an oven at 80 °C. The drop-casting was repeated 4 times; 2 drops on the front side and 2 drops on the back side. The electrode was then transferred to a muffle furnace. Ramp rates for a furnace were set to be 10 °C/min from room temperature to 400 °C and dwelled for 5 hours. The MnO_x nanoparticle electrodes were naturally cooled to room temperature in the furnace. FeO_x nanoparticles were synthesized in a similar way, using iron acetate instead of manganese acetate precursor.

To perform the Ir decoration, typically, four MnO_x/CP electrodes were attached in a beaker using Kapton tape. The beaker was then placed in a 50 °C water bath. 40 mL of 0.1 mM K_2IrCl_6 aqueous solution was prepared separately in a conical centrifuge tube and the temperature of the solution was adjusted to 50 °C. We noted that the temperature of the iridium precursor-containing solution should be adjusted before adding it to the beaker. The solution was stirred by a mini magnetic bar during the decoration process (Figure S1). After 30 min, the electrodes were removed from the beaker, gently rinsed with de-ionized water several times, and dried in the 80 °C oven for 30 min. A similar procedure was used to make Pt_{single}-MnO_x. 100 μM $K_2Pt(IV)Cl_6$ aqueous solution was used and synthesis temperature and dwelling time were set to be 50 °C and 30 min, respectively.

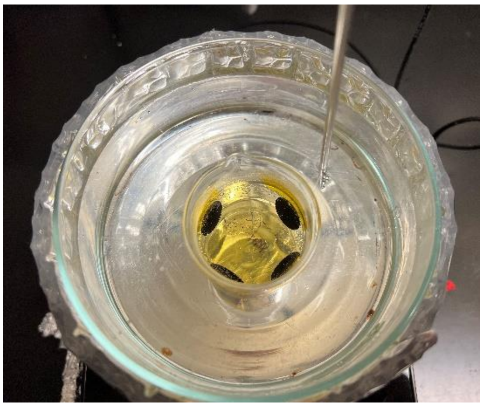

Figure S1. A water bath setup for Ir decoration on MnO_x/CP.

A.2.2. Ir_{few}-MnO_x synthesis

For the synthesis of Ir_{few}-MnO_x nanoparticles, the concentration of the precursor remained same and dwelling time at 50 °C was set to be 2 hours. After synthesis, the electrodes were gently rinsed with de-ionized water several times and dried in the 80 °C oven for at least 30 min.

A.2.3. $Ir_{few/cluster}$ – MnO_x nanoparticle synthesis

For the synthesis of $Ir_{few/cluster}$ -MnO_x nanoparticles, increased temperature and higher precursor concentration, 0.25 mM K_2IrCl_6 solution at 70 °C was used. Dwelling time at 70 °C was set to be 30 min.

A.2.4. $Ir_{cluster}$ -MnO_x nanoparticle synthesis

Sub-2-nm-sized iridium clusters were formed on MnO_x nanoparticles when we used 0.25 mM K_2IrCl_6 solution at 70 °C with 2h of dwelling time. Compared to $Ir_{few/cluster}$–MnO_x nanoparticle, the portion of clustered iridium particles on the MnO_x were significantly higher, which was confirmed by HR-TEM.

A.3. TEM analysis

High-resolution transmission electron microscopy (HR-TEM) images and scanning transmission electron microscopy (STEM) images were obtained using a high-resolution transmission electron microscope (JEOL ARM 200F, Japan) with an acceleration voltage of 200 kV. For TEM analysis, dispersed M_1-M_2O_x nanocatalysts were prepared on the ITO glass electrode. After heteroatom decoration, synthesized M_1-M_2O_x catalysts were carefully collected with a razor blade in an Eppendorf tube and dispersed in ethanol with the help of sonication. The ethanol solution was dropped on the TEM grid and dried in an oven under 80 °C before analysis.

A.4. X-ray photoelectron spectroscopy

XPS sample preparation and characterization. After electrolysis, the electrodes were rinsed with acetonitrile several times and dried under ambient air overnight. The electrodes were cut into small pieces and affixed to an XPS sample holder using conductive Cu tape. Then, the holder was transferred to the XPS sample introduction chamber. XPS spectra were collected using a Physical Electronics Versaprobe II X-ray Photoelectron Spectrometer and the data were analyzed with Casa XPS software.

B. Electrochemical study

B.1. Electrochemical methods

Electrochemical experiments were conducted with a sandwich-type one-compartment electrochemical cell. Platinum foil and M_1-M_2O_x loaded carbon paper were used as the counter and working electrode, respectively. An Ag/AgCl electrode (3.4 M KCl leak-free 2.0 mm diameter, Innovative Instruments) was used as the pseudo-reference electrode, and aluminum foil was used as the current collector. Hydrophobic carbon paper was placed behind the nanoparticle-loaded carbon paper to prevent leakage of the solvent. Acetonitrile

with 0.11 M $TBABF_4$ was used as the solvent with varying concentrations of cyclooctene and water. The total volume of electrolyte was set to be 4 mL. The resistance value at open circuit potential (OCP) was measured by electrochemical impedance spectroscopy techniques. In Figure 4A & B, the potentials were 100% iR compensated (85% automatic compensation with the last 15% manually compensated for potential-dependence experiments), and in other experiments, the potentials were 85% IR-compensated automatically. The electrochemical measurements were conducted with VMP3 Multi-channel potentiostat from BioLogic. For the potential dependent and water-dependent experiments, 10 coulombs were passed. For the substrate-dependent analysis, 5 C was passed for 10, 25, and 50 mM cyclooctene, and 10 C were passed for 100 and 200 mM cyclooctene.

The Ag/AgCl pseudo-reference electrode was calibrated against the ferrocene/ferrocenium (Fc/Fc^+) redox potential, which was obtained by averaging the potential of the oxidative and reductive peaks from the cyclic voltammetry with a scan rate of 50 mV/s. The calibration solutions were prepared by adding ~5 mM of ferrocene to electrolyte solutions (0.11 M $TBABF_4$, 0.2 M cyclooctene in acetonitrile) with varying water concentrations. Most of the experiments used 10 M water electrolyte, except for the data in Figure 4C and D. Vapor-liquid equilibrium was used to measure the water activity for different concentrations of water in the electrolytes. A previously reported headspace gas sampling protocol was employed to generate Figure S2.[2]

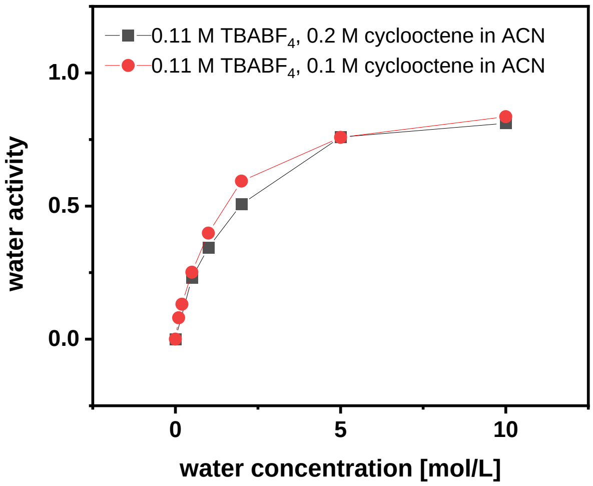

Figure S2. Measured water activity for different water concentrations in the electrolyte.

All cell parts including the counter electrode were washed with acetone and MilliQ water after every experiment and sonicated for at least 15 minutes in 10% nitric acid aqueous solution to remove any contaminants. The cell parts were washed with MilliQ water again after the sonication and oven-dried at 80 °C for at least 20 minutes before cell assembly to remove any residual moisture.

B.2. Long-term chronoamperometry

Long-term electrolysis with a small amount of stagnant electrolyte can cause the accumulation of products, which can be detrimental to the catalyst performance. Therefore we refreshed the electrolyte (4.0 mL) every ~15 min during the chronoamperometry experiment for over two hours. When switching the electrolyte, the electrolysis was paused and restarted within 1 min. The potential-time plot in Figure S3 includes those interval times as well as the electrolysis times.

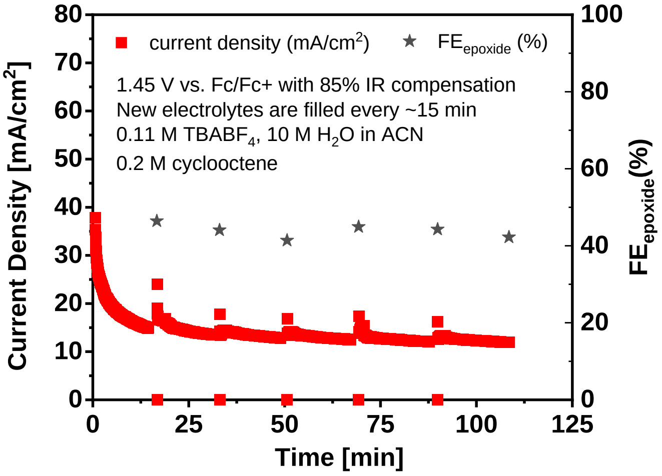

Figure S3. Long-term chronoamperometry experiment at 1.45 V vs. Fc/Fc$^+$ (85% iR compensation) with 0.11 M $TBABF_4$, 10 M H_2O, 0.2 M cyclooctene in acetonitrile electrolyte, which is refreshed every ~15 minutes.

C. Product analysis

After the electrolysis, post-electrolysis electrolytes were collected using glass pipette. The collected electrolyte was often less than 4 mL, so ~ 0.2 mL of acetonitrile was added to the electrolyte chamber, rinsed the chamber, and then added the solution to the post-electrolysis electrolyte until the total volume of collected electrolyte becomes 4 mL. We sampled 2 mL of the collected electrolyte, and ~ 13 mL of additional water, ~ 5 mL of hexane, and the internal standard 1,3,5-trimethoxybenzene were added to the electrolyte. Cyclooctene, its oxidation products, and the internal standard moved to the hexane layer after extraction with the help of centrifugation at 3000 rpm for 30 seconds. Extraction with hexane was required to exclude $TBABF_4$ that can overlap with the products on ^{1}H-NMR spectra. Hexane extraction was performed three more times (~ 20 mL of hexane was used in total), and the combined hexane solution was quantified using ^{1}H-NMR. The ^{1}H-NMR spectra were measured on a Bruker Avance Neo spectrometer operating at 500.34 MHz with solvent suppression of the three largest peaks from hexane. Samples were prepared by combining 50 μL of the extracted hexane layer with a 600 μL $CDCl_3$. The ^{1}H probe was automatically tuned, the lock was achieved on the $CDCl_3$ in the sample, and gradient shimming and auto-gain were used. 32 scans were collected per sample, and relaxation delay was set to 6 seconds. Products were quantified by comparing the areas of the peaks of interest to those of

the internal standard, 1,3,5-trimethoxybenzene. Auto phase correction and baseline correction with Whittaker Smoother method from the MestReNova software was performed on the spectra before the integration of each peak.

D. Supplementary Tables and Figures

Table S1. Derivation of the standard electrode potential for the transition from MnO_2 to Mn_3O_4. The values for the reaction [1] to [4] are from the reference 19 of the manuscript.

Reaction	Index / calculation	ΔG ($kJ/mol_{reaction}$)	Potential vs. SHE (V)
$Mn^{3+} + e^- \rightarrow Mn^{2+}$	[1]	-150.5	1.56
$MnO_2(s) + 4H^+ + e^- \rightarrow Mn^{3+} + 2H_2O$	[2]	-104.2	1.08
$3Mn_2O_3(s) + 2H^+ + 2e^- \rightarrow 2Mn_3O_4(s) + H_2O$	[3]	-159.8	0.828
$2MnO_2(s) + 2H^+ + 2e^- \rightarrow Mn_2O_3(s) + H_2O$	[4]	-188.0	0.974
$2MnO_2(s) + Mn^{2+} + 2e^- \rightarrow Mn_3O_4(s)$	[([3]+3*[4])-2*([1]+[2])]/2	-107.1	0.555

Table S2. Metal concentrations in post-galvanic replacement solutions

	lost Ir in solution (mmol/L)	Mn in solution (mmol/L)
Ir_{single}-MnO_x synthesis condition<br>(0.1 mM Ir precursor at 50 °C for 30 min)	0.013974	0.013119
$Ir_{cluster}$-MnO_x synthesis condition<br>(0.25 mM Ir precursor at 70 °C for 120 min)	0.059388	0.028919

Table S3. EXAFS fitting parameters of MnO_x and Ir_{single}-MnO_x to Mn_3O_4

	Bond type	N	S_0^2	$\frac{Mn_{tet}}{Mn_{oct} + Mn_{tet}}$	σ^2	ΔE_0 (eV)	R_{eff} (Å)	ΔR (Å)	R-factor
MnO_x	Mn_{tet}-O	4	0.85	0.3 ± 0.1	0.003±0.002	4.1 ± 1.5	2.0831	-0.03±0.02	0.03
	Mn_{oct}-O_{ax}	4					1.9597	-0.02±0.02	
	Mn_{oct}-O_{hor}	2					2.3217	-0.03±0.03	
Ir_{single}-MnO_x	Mn_{tet}-O	4	0.85	0.2 ± 0.1	0.004±0.001	3.8 ± 1.4	2.0831	-0.03±0.02	0.02
	Mn_{oct}-O_{ax}	4					1.9597	-0.02±0.02	
	Mn_{oct}-O_{hor}	2					2.3217	-0.03±0.02	

Table S4. ICP-OES result of the post-electrolysis electrolyte

Metal type	Initial metal amount on the Ir_{single}-MnO_x electrode (mmol)	Amount of metal in post-electrolysis solution (mmol)	Leached metal ratio (%)
Mn	0.002987	1.7831E-06	0.06
Ir	0.0001397	1.05476E-07	0.08

Table S5. The magnitude of Mn 3S Peak splitting in different catalysts

Sample	Magnitude of peak splitting (eV)
MnO_x	5.66
Post-electrolysis MnO_x	5.59
Ir_{single}-MnO_x	5.52
Post-electrolysis Ir_{single}-MnO_x	5.38
$Ir_{cluster}$-MnO_x	5.11
Pt_{single}-MnO_x	5.24

Table S6. Selectivity and total average current density with different catalysts

Catalyst	Potential	FE_epoxide	Total current density (mA/cm^2)
Pt_{single}-MnO_x	1.35 V vs. Fc/Fc+, 100% IR compensated	33.0%	13.6
FeO_x	1.4 V vs. Fc/Fc+, 85% IR compensated	10.85%	16.5
Ir_{single}-FeO_x	1.4 V vs. Fc/Fc+, 85% IR compensated	10.13%	14.7
FeO_x	1.45 V vs. Fc/Fc+, 85% IR compensated	12.16%	29
Ir_{single}-FeO_x	1.45 V vs. Fc/Fc+, 85% IR compensated	12.16%	23.8

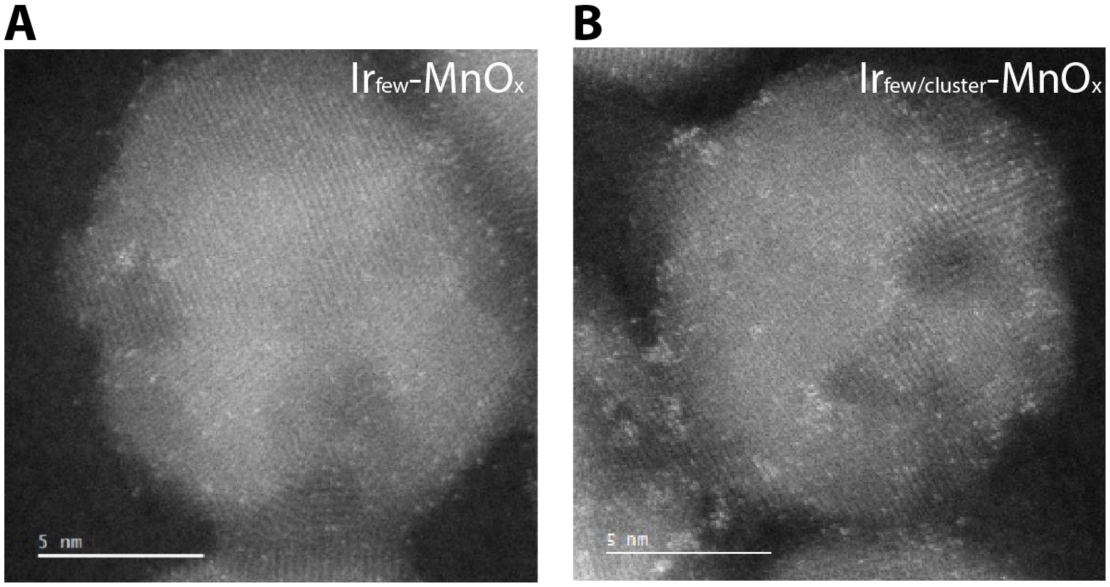

Figure S4. STEM images of **A)** Ir_{few}-MnO_x and **B)** $Ir_{few/cluster}$-MnO_x nanoparticles

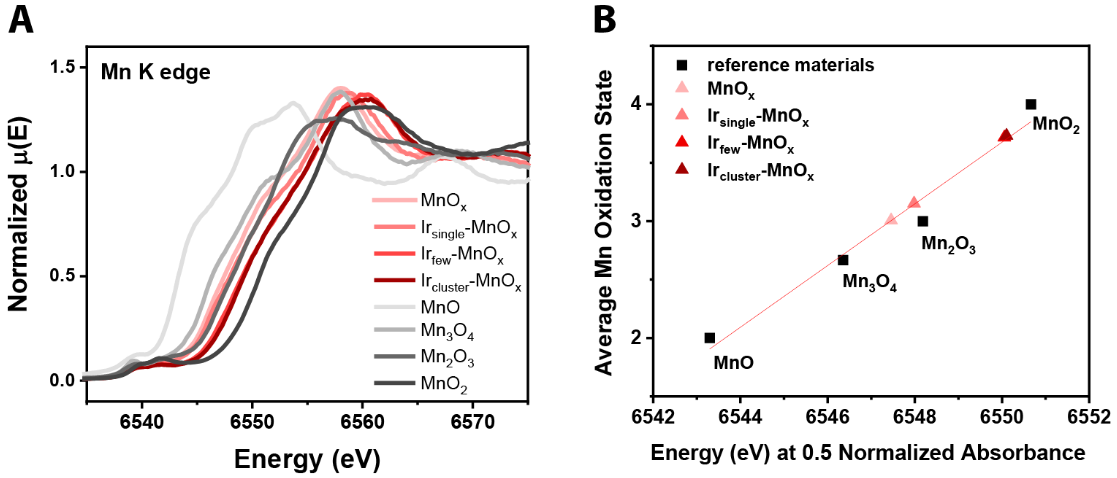

Figure S5. Mn K-edge XANES results of manganese oxide reference materials and the catalysts.

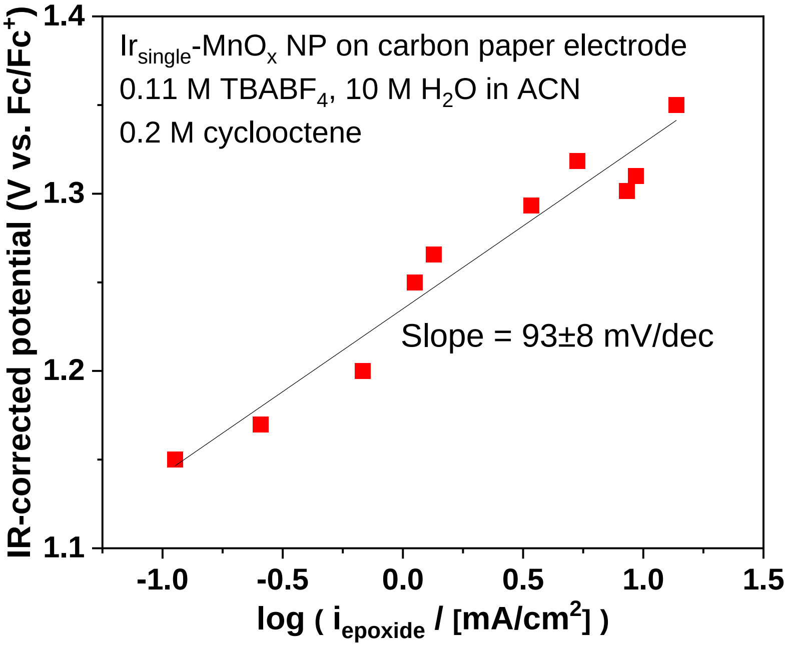

Figure S6. Tafel plot for cyclooctene formation with 10 M H_2O and 0.2 M cyclooctene.

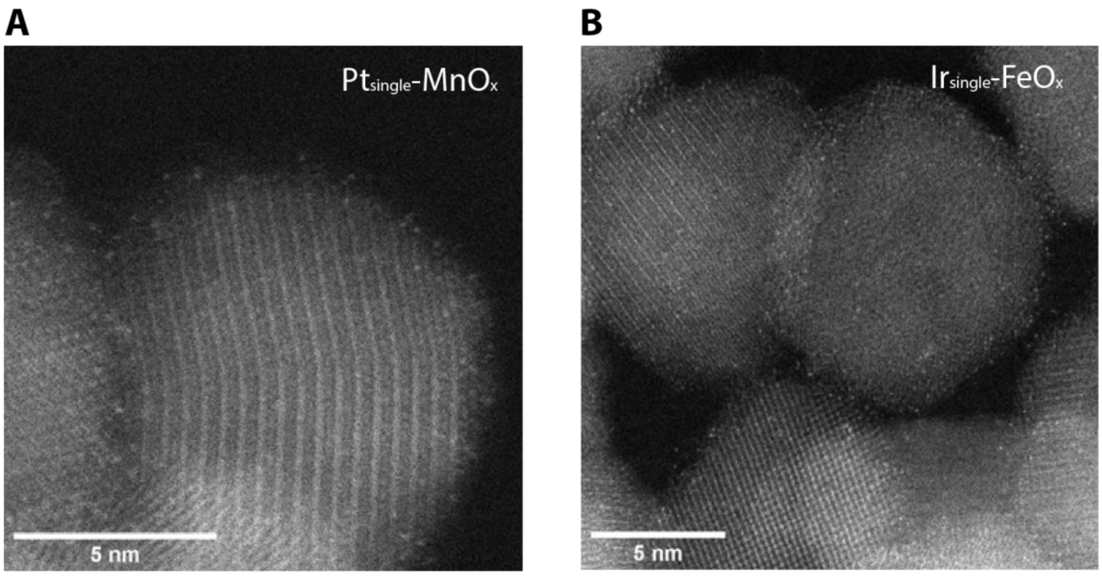

Figure S7. STEM images of **A)** Pt_{single}-MnO_x and **B)** Ir_{single}-FeO_x nanoparticles.

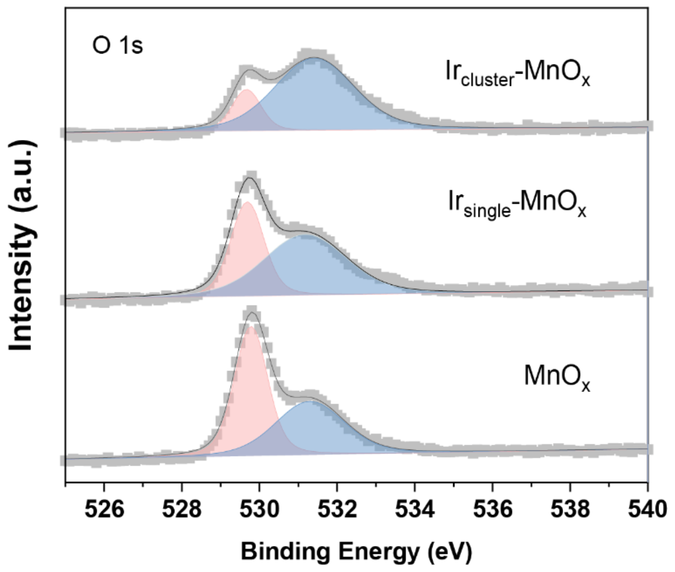

Figure S8. High-resolution O 1s XPS spectra of MnO_x, Ir_{single}-MnO_x, and $Ir_{cluster}$-MnO_x.

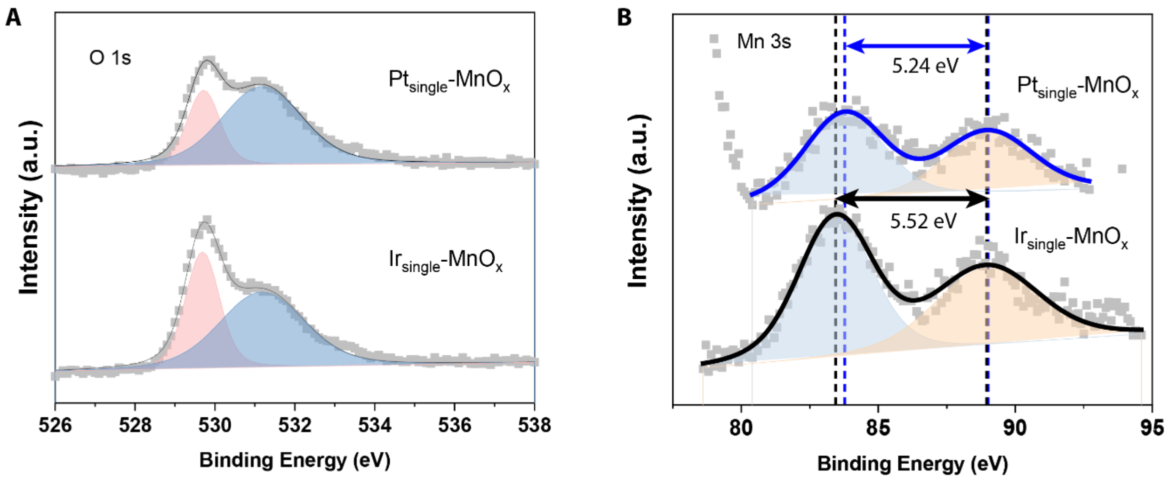

Figure S9. High-resolution A) O 1s and B) Mn 3s XPS spectra of Pt_{single}-MnO_x and Ir_{single}-MnO_x. High intensity data points at binding energies less than 80 eV for Pt_{single}-MnO_x are from Pt 4f signal.

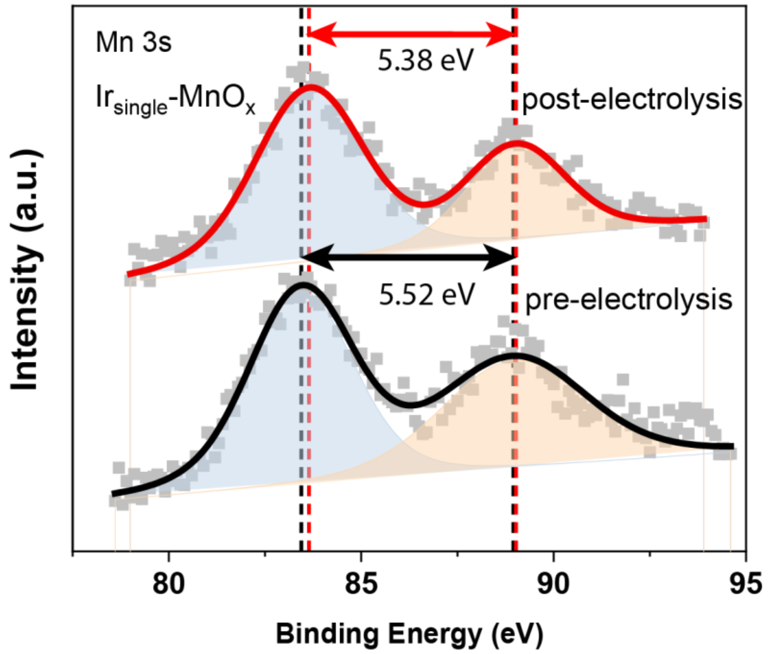

Figure S10. High-resolution Mn 3s XPS spectra of post- and pre-electrolysis Ir$_{single}$-MnO$_x$.

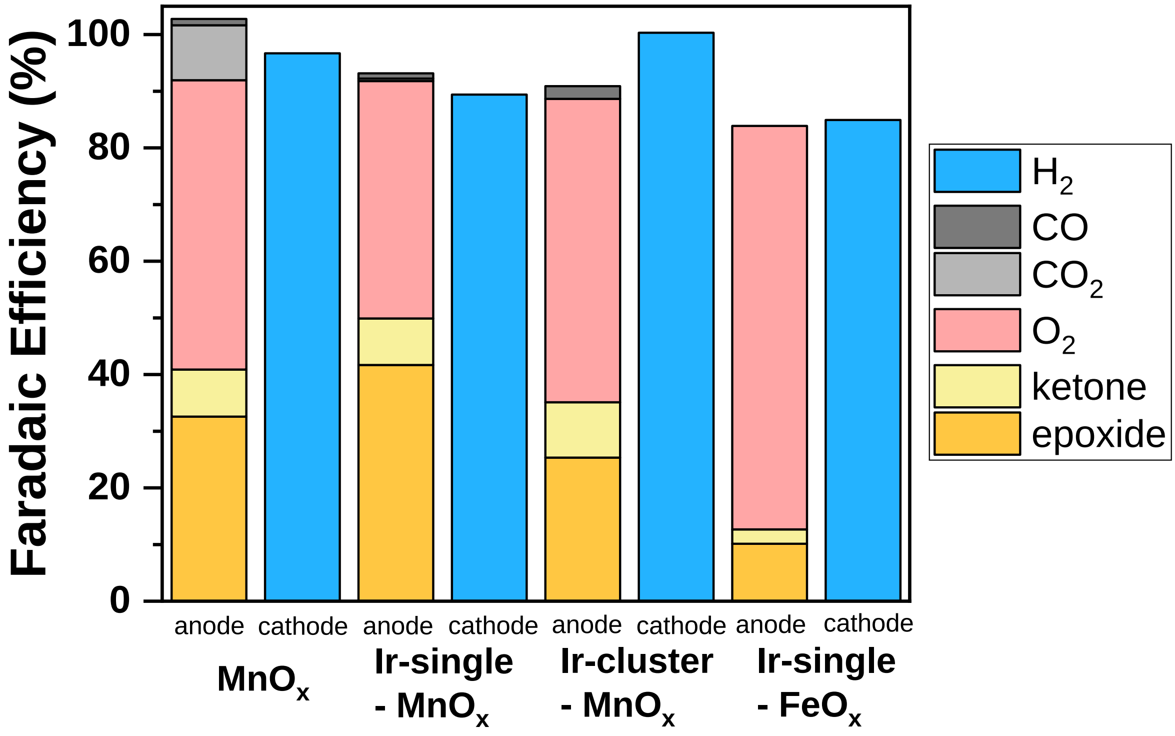

Figure S11. Product distributions with different catalysts based on Faradaic efficiencies at the anode and the cathode

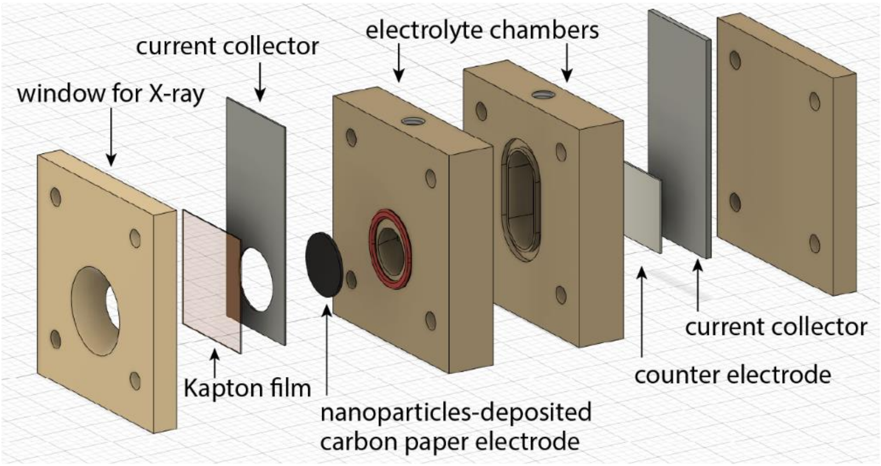

Figure S12. Electrochemical cell used for operando X-ray absorption spectroscopy.

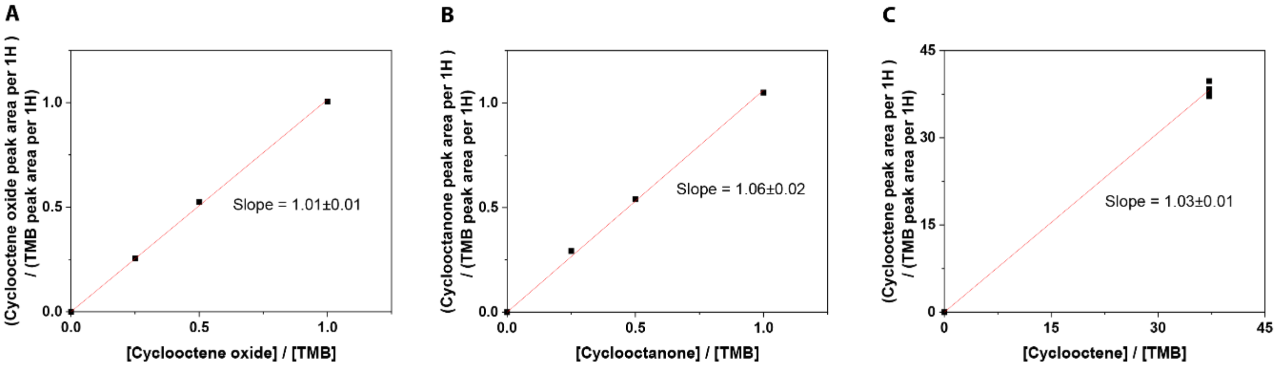

Figure S13. Calibration curves for the product and substrate ^{1}H-NMR peak area with respect to the internal standard peak area. Calibration solutions were prepared by adding known amounts of products and substrates with 0.11 M $TBABF_4$ and 10 M H_2O in acetonitrile solution, which represent representing typical experimental conditions. The products and substrates were extracted and analyzed by the method described in the section C.

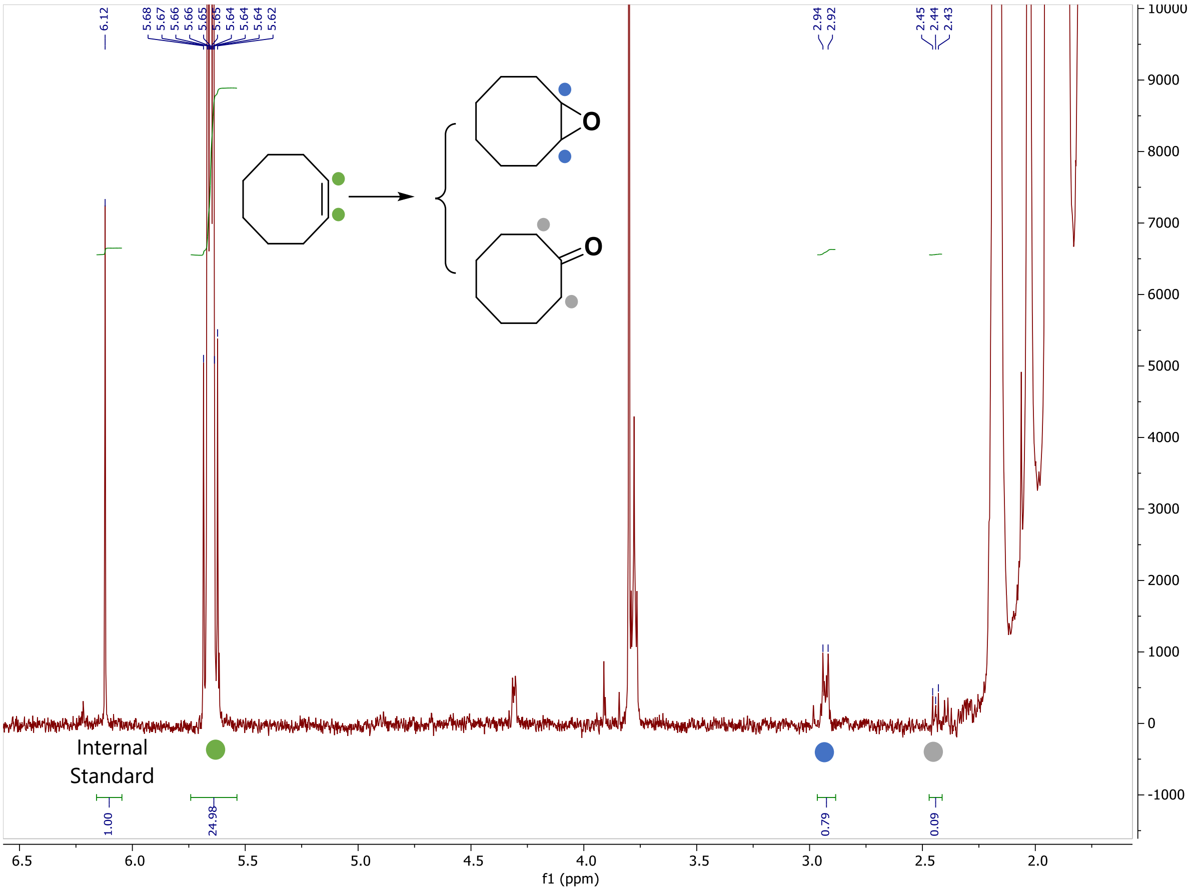

Figure S14. A representative ^{1}H-NMR spectrum of the extracted reaction mixture from the electrolysis using Ir_{single}-MnO_x as the anode catalyst at 1.3 V vs. Fc/Fc^{+}.

E. Discussion on the cyclooctene epoxidation mechanism

The proposed mechanism in our previous work includes chemical reactions as below.

$$\theta_O + \text{olefin} \underset{k_{-1}}{\overset{k_1}{\rightleftharpoons}} \theta + \text{epox}$$

$$\theta + H_2O \xrightarrow{k_2} \theta_{H_2O} + H_2O + e^- \ (RDS)$$

$$\theta_{H_2O} \underset{k_{-3}}{\overset{k_3}{\rightleftharpoons}} \theta_{OH} + H^+$$

$$\theta_{OH} \underset{k_{-4}}{\overset{k_4}{\rightleftharpoons}} \theta_O + e^- + H^+$$

With Langmuir isotherm assumptions, the density of different manganese active sites can be expressed as below.

$$\theta_O = \frac{k_{-1}}{k_1}\frac{a_{epox}}{a_{olefin}}\theta$$

$$\theta_{OH} = \frac{k_{-4}}{k_4} a_{H^+}\theta_O \exp(\frac{-FE}{RT}) = \frac{k_{-1}}{k_1}\frac{k_{-4}}{k_4}\frac{a_{H^+}a_{epox}}{a_{olefin}}\theta \exp(\frac{-FE}{RT})$$

$$\theta_{H_2O} = \frac{k_{-3}}{k_3} a_{H^+}\theta_{OH} = \frac{k_{-1}}{k_1}\frac{k_{-3}}{k_3}\frac{k_{-4}}{k_4}\frac{a_{H^+}^2 a_{epox}}{a_{olefin}}\theta \exp(\frac{-FE}{RT})$$

The total active site density can be expressed as below.

$$\theta_{all} = \theta + \theta_O + \theta_{OH} + \theta_{H_2O}$$
$$= \theta(1 + \frac{k_{-1}}{k_1}\frac{a_{epox}}{a_{olefin}} + \frac{k_{-1}}{k_1}\frac{k_{-4}}{k_4}\frac{a_{H^+}a_{epox}}{a_{olefin}}\exp(\frac{-FE}{RT}) + \frac{k_{-1}}{k_1}\frac{k_{-3}}{k_3}\frac{k_{-4}}{k_4}\frac{a_{H^+}^2 a_{epox}}{a_{olefin}}\exp(\frac{-FE}{RT}))$$

Then, the reaction rate towards cyclooctene oxide can be expressed as below. It is assumed that the cathodic reaction in the rate-determining step is negligible since anodic potential is applied.

$$\frac{i_{products}}{2F} = k_2 a_{H_2O}\theta \exp\left(\frac{(1-\beta)FE}{RT}\right)$$
$$= \frac{k_2 a_{H_2O}\theta_{all}\exp\left(\frac{(1-\beta)FE}{RT}\right)}{\left(1 + \frac{k_{-1}}{k_1}\frac{a_{epox}}{a_{olefin}} + \frac{k_{-1}}{k_1}\frac{k_{-4}}{k_4}\frac{a_{H^+}a_{epox}}{a_{olefin}}\exp(\frac{-FE}{RT}) + \frac{k_{-1}}{k_1}\frac{k_{-3}}{k_3}\frac{k_{-4}}{k_4}\frac{a_{H^+}^2 a_{epox}}{a_{olefin}}\exp(\frac{-FE}{RT})\right)}$$

If we assume that θ_O is the resting state and the population of it during the reaction cycle outcompetes that of other surface species, other terms in the dominator except for the second term can be neglected, giving a theoretical Tafel slope of 118 mV/dec and first-order dependencies on cyclooctene and water activity. However, when other surface species are present in comparable amounts to the metal-oxo species, it is difficult to expect a cardinal Tafel slope, and non-linearity would be observed in the Tafel plot. Therefore, the observed Tafel slope of 93±8 mV/dec (Figure S4) for Ir_{single}-MnO_x catalysts toward cyclooctene epoxidation may reflect a more distributed surface species population. The order dependencies can be complicated as well when there are no dominant species. However, in our system, the given rate equation indicates that the first order in the water activity would be conserved in any case, and first-order in cyclooctene would be conserved as well if the sum of occupied sites is much bigger than the number of vacant sites ($\theta << \theta_O + \theta_{OH} + \theta_{H_2O}$).

Another complication in this system is that both iridium and manganese can be the active site for cyclooctene epoxidation. Iridium sites could have larger reaction constants (k_1, k_2, k_3, k_4) than manganese which would result in a high epoxidation rate. Manganese leaching could create more active sites (θ_{all}) on the surface, which would contribute to the increase in cyclooctene epoxidation rate. Moreover, we should consider competing reactions to account for trends in selectivity. Competition between olefin epoxidation and water oxidation can be governed by the following two reactions.

$$\theta_O + \text{olefin} \underset{k_{-1}}{\overset{k_1}{\rightleftarrows}} \theta + \text{epox}$$

$$\theta_O + H_2\text{O} \underset{k_{-5}}{\overset{k_5}{\rightleftarrows}} \theta_{OOH} + e^- + H^+$$

If the iridium active sites and/or modified manganese active sites in Ir_{single}-MnO_x have larger k_1 / k_5 ratio compared to the manganese active sites in MnO_x, the selectivity toward epoxidation will increase. On iridium clusters, oxygen evolution could exhibit a binuclear oxygen-coupling mechanism, which could decrease selectivity toward epoxidation if the new route for oxygen evolution is favored.

References

(1) Jin, K.; Maalouf, J. H.; Lazouski, N.; Corbin, N.; Yang, D.; Manthiram, K. Epoxidation of Cyclooctene Using Water as the Oxygen Atom Source at Manganese Oxide Electrocatalysts. *J. Am. Chem. Soc.* **2019**, *141* (15), 6413–6418. https://doi.org/10.1021/jacs.9b02345.

(2) Williams, K.; Limaye, A.; Weiss, T.; Chung, M.; Manthiram, K. Accounting for Species' Thermodynamic Activities Changes Mechanistic Interpretations of Electrochemical Kinetic Data. **2022**. https://doi.org/10.26434/CHEMRXIV-2022-VK5Z9.
